# Acute exposure to air pollution particulate matter aggravates experimental myocardial infarction in mice by potentiating cytokine secretion from lung macrophages

**DOI:** 10.1007/s00395-016-0562-5

**Published:** 2016-05-30

**Authors:** Timoteo Marchini, Dennis Wolf, Nathaly Anto Michel, Maximilian Mauler, Bianca Dufner, Natalie Hoppe, Jessica Beckert, Markus Jäckel, Natalia Magnani, Daniel Duerschmied, Deborah Tasat, Silvia Alvarez, Jochen Reinöhl, Constantin von zur Muhlen, Marco Idzko, Christoph Bode, Ingo Hilgendorf, Pablo Evelson, Andreas Zirlik

**Affiliations:** Atherogenesis Research Group, Cardiology and Angiology I, University Heart Center, University of Freiburg, Hugstetterstrasse 55, 79106 Freiburg, Germany; Instituto de Bioquímica y Medicina Molecular (IBIMOL UBA-CONICET), Facultad de Farmacia y Bioquímica, Universidad de Buenos Aires, Buenos Aires, Argentina; Department of Pneumology, University of Freiburg, Freiburg, Germany; School of Science and Technology, National University of General San Martín, Buenos Aires, Argentina

**Keywords:** Myocardial infarction, Inflammation, ROFA, Particulate matter, Monocytes

## Abstract

Clinical, but not experimental evidence has suggested that air pollution particulate matter (PM) aggravates myocardial infarction (MI). Here, we aimed to describe mechanisms and consequences of PM exposure in an experimental model of MI. C57BL/6J mice were challenged with a PM surrogate (Residual Oil Fly Ash, ROFA) by intranasal installation before MI was induced by permanent ligation of the left anterior descending coronary artery. Histological analysis of the myocardium 7 days after MI demonstrated an increase in infarct area and enhanced inflammatory cell recruitment in ROFA-exposed mice. Mechanistically, ROFA exposure increased the levels of the circulating pro-inflammatory cytokines TNF-α, IL-6, and MCP-1, activated myeloid and endothelial cells, and enhanced leukocyte recruitment to the peritoneal cavity and the vascular endothelium. Notably, these effects on endothelial cells and circulating leukocytes could be reversed by neutralizing anti-TNF-α treatment. We identified alveolar macrophages as the primary source of elevated cytokine production after PM exposure. Accordingly, in vivo depletion of alveolar macrophages by intranasal clodronate attenuated inflammation and cell recruitment to infarcted tissue of ROFA-exposed mice. Taken together, our data demonstrate that exposure to environmental PM induces the release of inflammatory cytokines from alveolar macrophages which directly worsens the course of MI in mice. These findings uncover a novel link between air pollution PM exposure and inflammatory pathways, highlighting the importance of environmental factors in cardiovascular disease.

**Electronic supplementary material:**

The online version of this article (doi:10.1007/s00395-016-0562-5) contains supplementary material, which is available to authorized users.

## Introduction

Cardiovascular disease represents the leading cause of mortality worldwide [26]. Chronic and acute inflammation triggered by traditional and non-traditional risk factors has been identified as the driving force behind cardiovascular pathologies including atherosclerosis and myocardial infarction (MI) [14, 31, 49]. In fact, environmental factors, such as air pollution, strongly influence initiation and outcome of cardiovascular disease [32]. Accordingly, numerous epidemiological studies identified exposure to environmental particulate matter (PM)—one of the main components of what is widely referred to as ‘air pollution’—as a major cause of increased mortality from MI [6]. Particularly, an acute exposure to PM elevates the risk of MI within a few hours [39]. Following MI, increased risk of death, progression to heart failure, and subsequent MI have been associated with PM exposure in humans [52]. Moreover, daily changes in airborne PM levels correlate with increased hospitalization due to MI, heart failure, arrhythmias, and stroke [22]. In cumulative analyses, up to 80 % of the increase in PM-associated mortality was caused by cardiovascular complications, while respiratory complications accounted for only 20 % of total mortality, indicating that cardiovascular, but not respiratory disease is the main complication of air pollution [4]. In line with these findings, exposure to PM accelerated experimental atherosclerosis in hyperlipidemic rabbits [51] and worsened cardiac function in previous animal studies [28].

Based on the observation that PM inhalation increases the levels of circulating pro-inflammatory cytokines, inflammation may be the cause of amplified cardiovascular mortality in PM-exposed individuals [29, 40]. However, neither the underlying mechanisms of the proposed inflammatory response, nor the direct participation of PM in experimental MI has been tested so far. To interrogate a potential direct functional link between PM exposure and MI, we have hypothesized that PM—by its surrogate Residual Oil Fly Ash (ROFA)—directly modulates traits of experimental MI in mice.

## Methods

An expanded Methods section is available in the online-only Data Supplement.

### Particulate matter

ROFA particles were collected from Boston Edison Co., Mystic Power Plant, Mystic, CT, US, burning low-sulfur residual oil (No. 6 fuel oil) and were kindly provided by Dr. J. Godleski (Harvard School of Public Health, Harvard University, Boston, MA, US). ROFA samples were extensively characterized regarding their chemical composition and particle size. Vanadium, nickel, and iron are the predominant metals present as water-soluble sulfates in ROFA. Their particle mean aerodynamic diameter is 2.06 ± 1.57 μm [8, 38]. ROFA containing suspensions were freshly prepared by suspending particles in sterile saline solution at 5 mg/mL, followed by incubation in an ultrasonic water bath for 5 min before use.

### Animal protocols

Male 8-week-old wild type or lymphocyte-free Rag1-deficient (Rag1^−/−^) mice on a C57BL/6J background (Jackson Laboratories) were exposed to a suspension of ROFA particles in saline (1 mg/kg body weight) or the same volume of sterile saline solution (control group) by intranasal (i.n.) instillation as previously described [42]. MI was induced by permanent ligation of the left anterior descending coronary artery (LAD) [20]. All experimental protocols were approved by the Animal Ethics Committee of the University of Freiburg, Germany. Every procedure was carried out in accordance with institutional guidelines.

### Histology

Seven days after MI, hearts were excised, embedded in Tissue-Tek O.C.T. compound (Sakura Finetek), frozen, and sectioned into 6 μm slices. Infarct area was demarked by Masson’s Trichrome staining. The anti-CD11b antibody clone M1/70 (BD Biosciences) was used as a pan-myeloid cell marker in immunohistochemistry.

### Flow cytometry

Cell suspensions were stained for flow cytometry as previously described [47] and acquired on a Canto II cytometer (BD Biosciences). Antibodies were used as indicated. For intracellular cytokine staining, leukocytes were fixed and permeabilized by the Cytofix/Cytoperm kit (BD Biosciences) and incubated with fluorochrome-conjugated anti-TNF-α, anti-IL-6, and anti-MCP-1 antibodies (eBioscience). Data were analyzed with FlowJo (TreeStar). To quantify leukocytes resident in the heart, infarcted myocardial tissue was excised, weighted, digested, and the obtained cell suspensions were analyzed as previously described [20]. Specifically, cardiac monocytes were identified as CD45^+^, Lin^−^ (Lin = CD3, CD19, CD49b, Ly6G, NK1.1), CD11b^+^, F4/80^low^, MHCII^low^, CD11c^low^, CD68^low^, CD115^+^ cells, subdivided into Ly6C^high^ and Ly6C^low^ subsets; neutrophils were identified as CD45^+^, Lin^+^, CD11b^+^, MHCII^low^, CD11c^low^, SSC^high^, Ly6C^int^ cells; cardiac macrophages were identified as CD45^+^, Lin^−^, CD11b^+^, F4/80^high^ (MHCII^+^, CD68^high^) cells. Alveolar macrophages were identified in bronchoalveolar lavage (BAL) samples as CD45^+^, CD11b^low^, Siglec-F^+^, CD11c^+^, CD64^+^, F4/80^int^ cells (Supplemental Fig. 2).

### Intravital microscopy

Three hours after the exposure to ROFA particles, mice were anesthetized and leukocytes were fluorescently labeled by a retro-orbital injection of rhodamine. A loop of ileum was exteriorized and intravital microscopy was performed on mesenteric veins as previously described [47]. Rolling leukocyte flux was defined as the number of leukocytes moving at a lower velocity than erythrocytes. Adherent leukocytes were defined as cells that remained stationary for at least 30 s. Injection of 200 ng murine TNF-α i.p. served as positive control.

### Sterile peritonitis

One hour after ROFA exposure, C57BL/6J mice received an i.p. injection of 2 ml 4 % thioglycollate broth. After 4 h, a peritoneal lavage was performed and peritoneal exudate cells (PECs) were quantified in a hemocytometer after red cell lysis.

### Cytokine levels

A cytometric bead array (CBA assay, BD Biosciences) was used to quantify cytokine levels in plasma, BAL, and cell culture supernatants according to manufacturer’s protocol.

### Endothelium activation markers

Plasma levels of soluble ICAM-1 (sICAM-1) and VCAM-1 (sVCAM-1) were measured by ELISA (R&D Systems). Alternatively, ICAM-1 and VCAM-1 expression was quantified on in vitro cultured mouse endothelial cells by immunohistochemistry as previously described [43].

### Conditioned plasma

Mouse endothelial cells from non-exposed C57BL/6J mice were isolated and cultured as previously described [47]. Neutrophils, monocytes, and macrophages were isolated by magnetic bead separation. Cells were incubated with plasma (1 % v/v) from saline- or ROFA-exposed mice, or with ROFA particles (1 μg/mL). As indicated, incubation was performed in the presence of a blocking anti-TNF-α antibody (10 μg/mL). After 24 h, cell culture supernatants were analyzed for cytokines, and cellular expression of adhesion molecules was analyzed by immunohistochemistry and flow cytometry as previously described [48].

### Macrophage depletion

Alveolar macrophages were depleted from C57BL/6J mice before ROFA exposure by an i.n. instillation of 50 μl dichloromethylene bisphosphonate (clodronate) liposomes (5 mg/mL) as previously described [53]. PBS-loaded liposomes were used as control. Alternatively, 200 μl clodronate liposomes were injected i.v. for systemic depletion of macrophages.

### Statistical analysis

Data are presented as mean ± SEM. Unpaired Student’s *t* test was used to analyze differences between two groups. ANOVA followed by Student–Newman–Keuls post hoc test was performed to evaluate differences between more than two groups. Statistical significance was considered at *p* < 0.05.

## Results

### Exposure to ROFA particles aggravates myocardial infarction

**Fig. 1:**
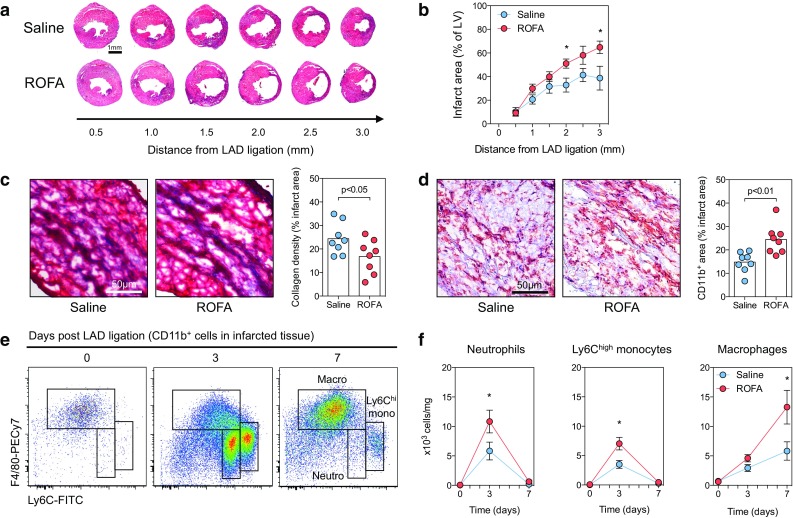
Exposure to ROFA particles aggravates MI. C57BL/6J mice were exposed to ROFA particles (1 mg/kg body weight) or an equal volume of sterile saline solution by i.n. instillation. MI was induced by permanent ligation of the left anterior descending coronary artery (LAD). After 7 days, infarct area was demarked and quantified by Masson’s Trichrome staining in cross sections of heart tissue at increasing distance from the site of coronary artery ligation (**a**, **b**). At 2 mm from the site of LAD ligation, collagen density was evaluated within the infarcted tissue (**c**), and myeloid cell infiltration was assessed by immunohistochemistry for the pan-myeloid marker CD11b (**d**). Myeloid cell infiltration was characterized by flow cytometry of digested hearts (**e**). Leukocyte populations of infarcted tissue from saline- and ROFA-exposed mice were quantified at the indicated time points after LAD ligation (**f**). Data are presented as mean ± SEM of at least seven mice per group. *p < 0.05

To test whether exposure to PM modulates MI, 8-week-old C57BL/6J mice were challenged with ROFA particles (1 mg/kg body weight) or an equal volume of sterile saline solution by i.n. instillation. After 1 day, MI was induced by permanent ligation of the left anterior descending coronary artery (LAD). Mice were subsequently instilled with either ROFA or saline solution on a daily basis. On day 7, hearts were excised and cross sections of the myocardium were stained with Masson’s Trichrome to depict infarction area (Fig. 1a). We detected a significant increase in infarction size by up to 67 ± 13 % (Fig. 1b) and decrease in collagen density (Fig. 1c) in ROFA-treated mice compared with respective control. These data indicate an adverse cardiac remodeling in ROFA-exposed mice. We also identified increased CD11b^+^ myeloid cell accumulation in hearts of ROFA-treated mice by immunohistochemistry (Fig. 1d). We observed no differences in overall survival (data not shown) or MI-associated arrhythmias in ROFA-exposed mice (Supplemental Fig. 1), but a slight increase in the duration of ventricular complexes 3 days after MI in this group (Supplemental Table 1). The latter is consistent with previous observations that ROFA exposure enhances the vulnerability to cardiac arrhythmias [46]. To further characterize the dynamics of myeloid cell infiltration to cardiac tissue, leukocyte populations in the heart were quantified by flow cytometry on days 0, 3, and 7 after LAD ligation (Fig. 1e). Following MI, ROFA-exposed mice showed enhanced accumulation of neutrophils, inflammatory Ly6C^high^ monocytes, and macrophages (Fig. 1f). Particularly, macrophage numbers increased by 125 ± 31 % in ROFA-exposed mice at day 7 (Fig. 1f). These results demonstrate that exposure to ROFA particles increases MI size and myocardial inflammatory cell accumulation, in particular that of macrophages.

### ROFA particles promote trafficking of inflammatory leukocytes by enhancing leukocyte activation

**Fig. 2:**
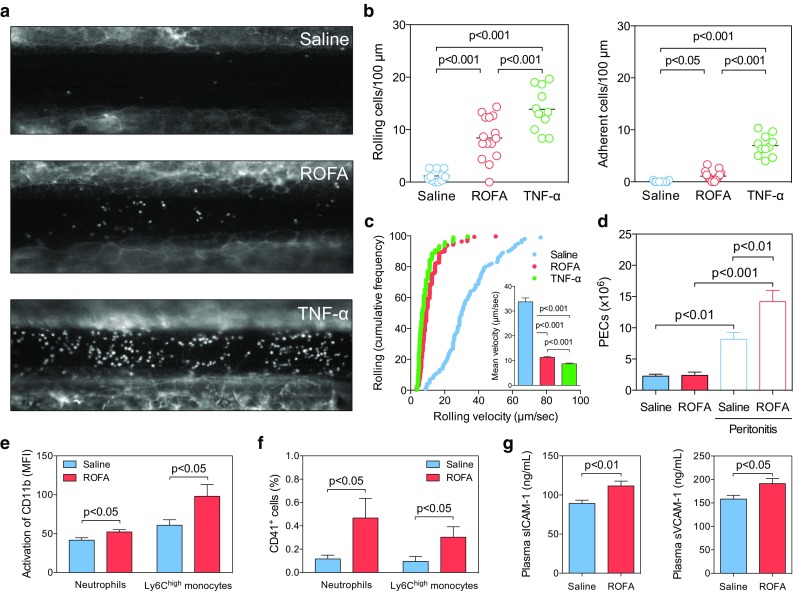
A single instillation of ROFA particles promotes adhesion and migration of pro-inflammatory leukocytes. C57BL/6J mice were treated with either saline, a suspension of ROFA particles in saline (1 mg/kg body weight), or murine TNF-α (200 ng i.p.) as positive control. After 3 h, leukocyte recruitment in mesenteric venules was assessed in intravital microscopy. Leukocytes were stained by rhodamine (**a**) and leukocyte rolling, adhesion (**b**), and cumulative frequency of the rolling velocity (**c**) were quantified. The inlay in **c** represents leukocyte mean rolling velocity. To evaluate whether ROFA treatment primes leukocytes to migrate, the number of cells residing in the peritoneal cavity (PECs) was quantified in saline- or ROFA-exposed mice. Leukocyte migration was forced by inducing sterile peritonitis by an i.p. injection of 4 % thioglycollate broth (**d**). To assess leukocyte activation, expression of the CD11b activation epitope (CRBM1/5, **e**) and formation of leukocyte-platelet aggregates (**f**) were quantified on myeloid cells by flow cytometry. Plasma markers of endothelial activation (**g**) were quantified by ELISA. Data are presented as mean ± SEM of at least 10 mice per group

To directly test whether an acute exposure to ROFA particles enhances inflammatory cell recruitment, a main pathomechanism in MI, we tested two in vivo models. First, we treated C57BL/6J mice by an i.n. instillation of ROFA particles (1 mg/kg body weight), saline solution, or an i.p. injection of murine TNF-α (200 ng), and monitored leukocyte recruitment in mesenteric venules by intravital microscopy (Fig. 2a). Interestingly, we observed a significant increase in the number of rolling and adhering leukocytes in ROFA-exposed mice (Fig. 2a, b, Supplemental Videos I–III). Accordingly, average rolling velocity was decreased after ROFA exposure (Fig. 2c). TNF-α i.p. treatment was used as a positive control and increased leukocyte rolling and adhesion, as well as decreased mean rolling velocity (Fig. 2c). Secondly, we tested whether ROFA exposure is capable of modulating leukocyte migration in vivo. Therefore, C57BL/6J mice were treated with ROFA as described above, and the number of cells accumulating in the peritoneal cavity was quantified. We did not observe relevant changes after ROFA exposure alone (Fig. 2d). However, after induction of sterile peritonitis by thioglycollate, simultaneous exposure to ROFA increased the number of peritoneal exudate cells (PECs) by 74 ± 12 % compared to saline (Fig. 2d), indicating that a ROFA exposure can boost, but not initiate leukocyte migration. To explore a potential mechanism, we assessed leukocyte activation by quantifying the abundance of the activation epitope CBRM1/5 on the leukocyte integrin CD11b/CD18 (Mac-1), an important mediator of leukocyte rolling and adhesion [10]. Interestingly, ROFA treatment significantly increased CD11b activation on circulating neutrophils and inflammatory Ly6C^high^ monocytes (Fig. 2e). Moreover, we observed a significant increase in neutrophil- and Ly6C^high^ monocyte-platelet aggregates in this group (Fig. 2f). Notably, plasma levels of the soluble fraction of the endothelial adhesion molecules ICAM-1 and VCAM-1 increased (Fig. 2g), suggesting that a ROFA exposure also induces endothelial cell activation. Taken together, these findings indicate that the exposure to ROFA particles induces inflammatory leukocyte recruitment, possibly by the up-regulation of adhesion molecules on endothelial and circulating myeloid cells.

### Activation of leukocytes and endothelial cells following ROFA exposure can be reversed by blockade of pro-inflammatory cytokines

**Fig. 3:**
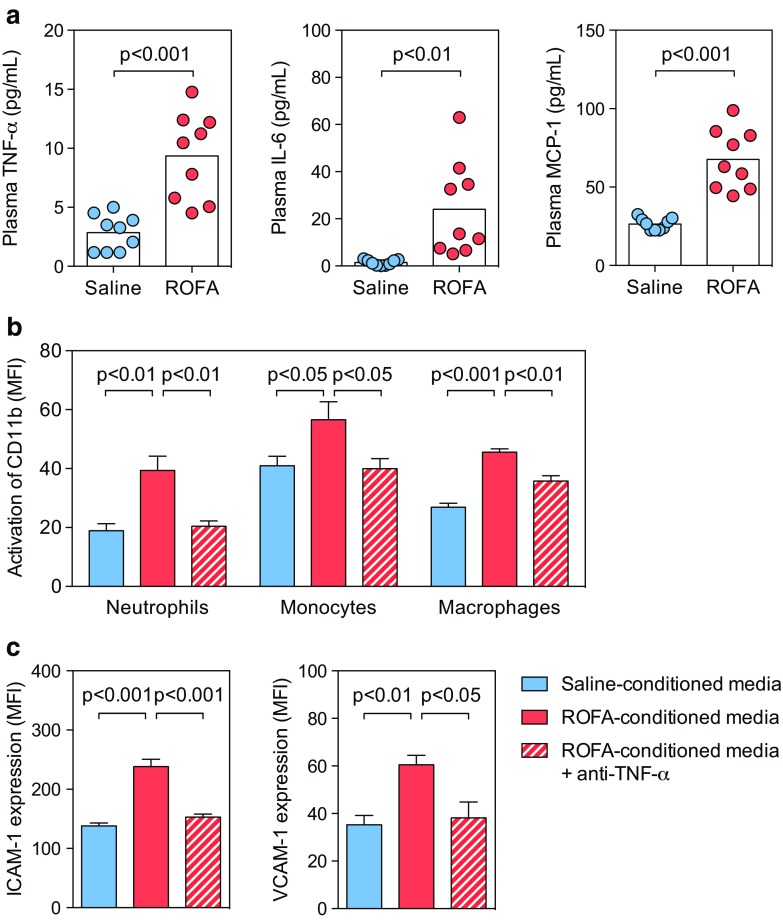
Activation of leukocytes and endothelial cells by ROFA is reversed by blockade of the pro-inflammatory cytokine TNF-α. C57BL/6J mice were exposed to saline or a suspension containing ROFA particles (1 mg/kg body weight) by i.n. instillation, and plasma levels of pro-inflammatory cytokines were quantified by a cytometric bead array (**a**). Isolated myeloid and endothelial cells were incubated in vitro with conditioned plasma (1 % v/v) from saline- or ROFA-exposed mice in the presence or absence of a blocking anti-TNF-α antibody (10 μg/ml). After 24 h, the abundance of the CD11b activation epitope CBRM1/5 was quantified by flow cytometry in myeloid cells (**b**). On endothelial cells, expression of ICAM-1 and VCAM-1 was determined by flow cytometry (**c**). Data are presented as mean ± SEM from at least nine mice per group (**a**) and from at least three independent experiments (**b**, **c**)

It has been suggested that exposure to PM increases the levels of circulating pro-inflammatory cytokines, but it is not known whether these cytokines are required for inflammatory leukocyte recruitment after PM inhalation [5]. Therefore, we hypothesized that cytokines circulating in the blood activate leukocytes and endothelial cells in our model. We first measured TNF-α, IL-6, and MCP-1 in the plasma of exposed mice. Consistent with previous reports, we detected elevated cytokine levels, such as TNF-α (an increase up to 327 ± 100 %) and IL-6 (an increase up to 1686 ± 469 %) compared with samples from control mice (Fig. 3a). Next, we tested the impact of cell culture media supplemented with conditioned plasma from saline- or ROFA-exposed mice (1 % v/v) to ex vivo cultures of isolated myeloid and endothelial cells. Supplementation of plasma from mice exposed to ROFA increased activation of leukocytes and endothelial cells in vitro, as assessed by CD11b activation and expression of endothelial adhesion factors (Fig. 3b, c). Interestingly, a neutralizing anti-TNF-α antibody reversed these effects. Similar results were obtained with a blocking anti-IL-6 antibody (data not shown). These findings indicate that pro-inflammatory cytokines circulating in the plasma after ROFA exposure increase activation of leukocytes and endothelial cells.

### ROFA particles do not directly activate endothelial or circulating myeloid cells

**Fig. 4:**
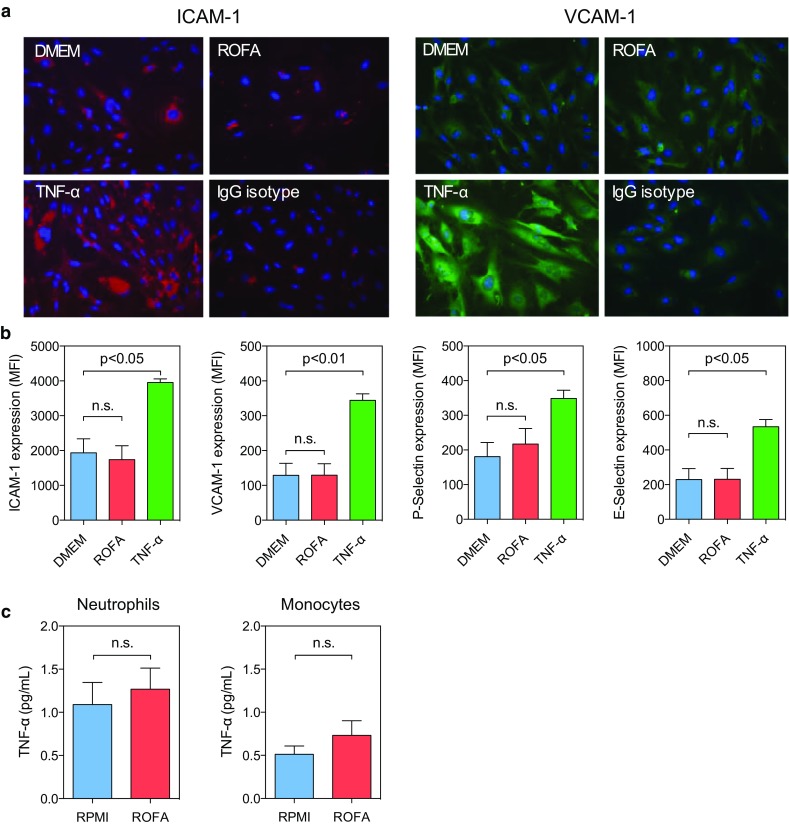
Direct exposure to ROFA particles does not activate endothelial or circulating myeloid cells. Endothelial cells were incubated in vitro with saline, ROFA particles (1 μg/mL), or TNF-α (10 ng/mL) as positive control. Expression of the adhesion molecules ICAM-1, VCAM-1, P-Selectin, and E-Selectin were quantified after 24 h by immunohistochemistry (**a**) as well as by flow cytometry (**b**). Isolated myeloid cells were incubated in vitro with ROFA particles (1 μg/mL), and TNF-α levels were measured in cell culture supernatants (**c**). IgG Isotype in panel **a** indicates the staining with an unspecific FITC- or TRITC-coupled isotype antibody. Data are presented as mean ± SEM of at least three independent experiments per group.* ns* indicates not significant

Nano-scale particles and soluble PM constituents can break through the respiratory epithelia and exert direct effects in systemic circulation within minutes to hours [33, 45]. To address whether ROFA particles circulating in the blood directly induce a cellular response, we challenged endothelial and circulating myeloid cells, such as neutrophils and monocytes, with a concentration of ROFA particles higher than expected after inhalation (1 μg/mL). Interestingly, exposure to ROFA particles had no effect on the expression of ICAM-1, VCAM-1, P-Selectin, or E-Selectin in cultured endothelial cells, as shown by immunohistochemistry and flow cytometry (Fig. 4a, b), while the addition of TNF-α strongly up-regulated these markers. Also, neutrophils and monocytes did not significantly increase TNF-α secretion after incubation with ROFA particles (Fig. 4c). These findings suggest that ROFA particles cannot directly activate endothelial or circulating myeloid cells.

### Exposure to ROFA particles induces the release of pro-inflammatory cytokines by alveolar macrophages

**Fig. 5:**
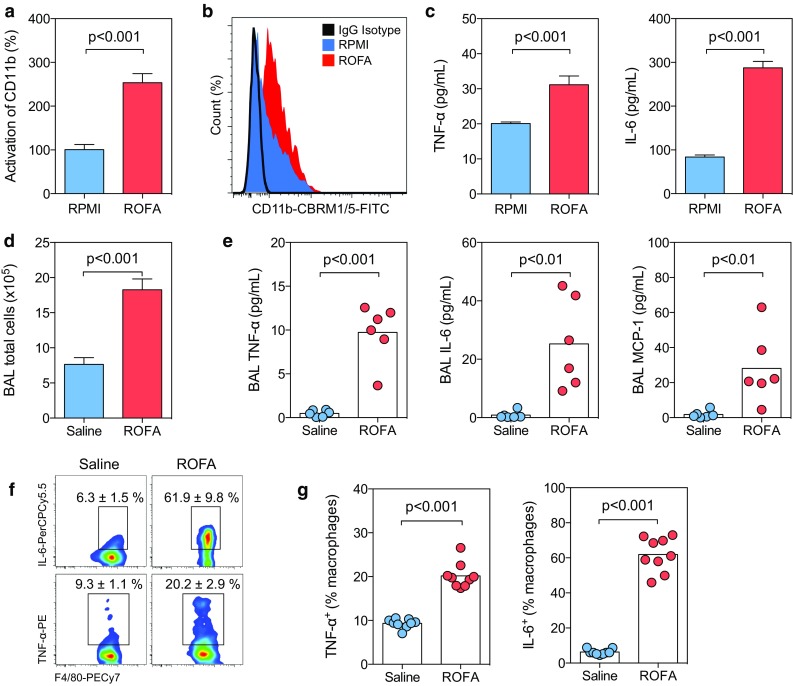
Exposure to ROFA particles induces the release of pro-inflammatory cytokines by alveolar macrophages. Macrophages isolated from the spleen were cultured in the presence of ROFA particles (1 μg/mL) or medium alone (RPMI). Cell activation was assessed by expression of the CD11b activation epitope CBRM1/5 (**a**, **b**). The concentration of pro-inflammatory cytokines was determined in cell culture supernatants (**c**). To quantify cytokine secretion in vivo, C57BL/6J mice were exposed to saline solution or ROFA particles (1 mg/kg body weight) by i.n. instillation. A bronchoalveolar lavage (BAL) was performed after 3 h and total cell numbers (**d**) and the concentration of pro-inflammatory cytokines were quantified (**e**). The BAL cell suspension was analyzed by flow cytometry. Alveolar macrophages were identified as CD45^+^, CD11b^low^, Siglec-F^+^, CD11c^+^, CD64^+^, F4/80^int^ cells (Supplemental Fig. 2), and intracellular cytokine staining was performed for TNF-α and IL-6 (**f**, **g**). Data are presented as mean ± SEM of at least three independent experiments (**a**–**c**) or at least six animals per group (**d**–**g**)

PM inhalation can trigger the accumulation of macrophages in the lung and the release of inflammatory cytokines [3, 17]. Therefore, we hypothesized that circulating pro-inflammatory cytokines detectable in blood may originate from alveolar macrophages. To give proof to this concept, we isolated macrophages from a splenocyte cell suspension by magnetic bead separation and incubated the obtained cells in RPMI media supplemented with either saline or ROFA particles. Macrophages exposed to ROFA showed increased CD11b activation (Fig. 5a, b) and augmented secretion of pro-inflammatory cytokines (Fig. 5c). Next, we confirmed that ROFA exposure was associated with an increase in total leukocyte count in bronchoalveolar lavage (BAL) as previously reported [17] (Fig. 5d). ROFA instillation strongly increased pro-inflammatory cytokine levels in BAL, including TNF-α and IL-6 (Fig. 5e). To track down the cellular origin of these cytokines, we performed intracellular cytokine staining in distinct leukocyte subpopulations by flow cytometry, and identified alveolar macrophages (CD45^+^, CD11b^low^, Siglec-F^+^, CD11c^+^, CD64^+^, F4/80^int^, Supplemental Fig. 2) as the primary source of these cytokines (Fig. 5f, g). Other cells, such as T- or B-lymphocytes, did not show relevant cytokine production (data not shown). Notably, Toll-like receptor (TLR) 4 expression in alveolar macrophages, but not in circulating myeloid cells, was increased (Supplemental Fig. 3), suggesting a functional role of TLR pathways in macrophage activation following ROFA exposure. These results indicate that alveolar macrophages are the predominant source of pro-inflammatory cytokines in the lung after exposure to environmental PM.

### Specific depletion of alveolar macrophages prevents ROFA-mediated inflammation

We hypothesized that ROFA exposure induces an inflammatory response driven by alveolar macrophages thereby impairing MI healing. To proof this concept in a loss-of-function approach, alveolar macrophages were depleted in our model by clodronate liposomes. Clodronate is a strongly hydrophilic bisphosphonate that—when delivered in liposomes—induces apoptosis in macrophages after internalization [44]. In a first approach, we injected clodronate liposomes i.v. to systemically deplete macrophages. PBS-loaded liposomes were used as control. After 24 h, i.v. clodronate pretreatment abolished ROFA-induced macrophage recruitment to the lung and reduced local and systemic cytokine levels in this group (Supplemental Fig. 4), indicating that macrophages are a relevant source of circulating pro-inflammatory cytokines. To specifically deplete alveolar macrophages, we delivered clodronate liposomes by i.n. instillation. This strategy was highly effective in depleting macrophages from the lung, but not from other locations (Supplemental Fig. 5). Alveolar macrophages were suppressed up to 5 days after a single dose of clodronate (Supplemental Fig. 6). Specific depletion of alveolar macrophages was effective in preventing both local and systemic cytokine release following ROFA exposure (Supplemental Fig. 7). To rule out that some of the effects induced by ROFA are at least partially mediated by the adaptive immune system, such as by lymphocytes [2, 41], we exposed lymphocyte-free Rag-1^−/−^ mice to ROFA particles. Notably, the cellular and inflammatory response resembled that of wild type mice (Supplemental Fig. 8), indicating that lymphocytes do not play a significant role in the acute inflammatory response initiated by ROFA exposure.

**Fig. 6:**
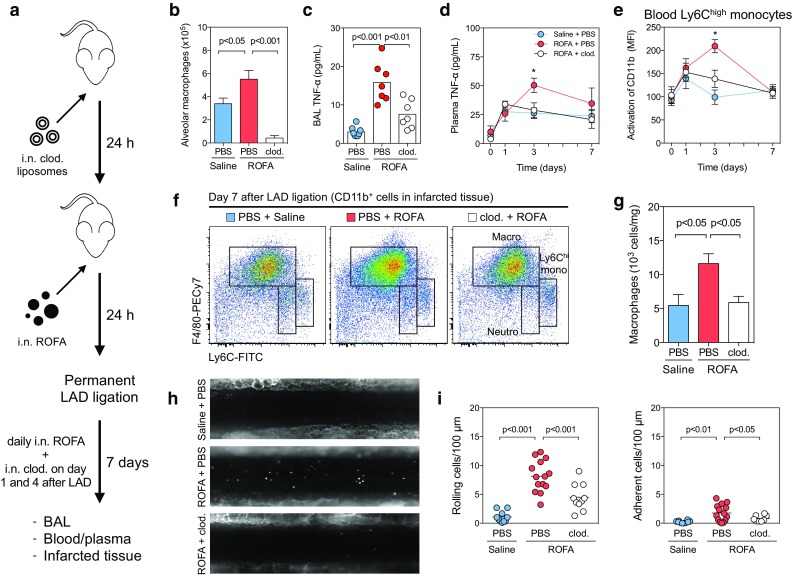
Specific depletion of alveolar macrophages prevents ROFA-mediated inflammation. To test their simultaneous effects, depletion of alveolar macrophages, ROFA exposure, and MI were performed gradually (**a**). After 7 days, numbers of alveolar macrophages in the BAL were determined by flow cytometry (**b**). TNF-α levels in BAL (**c**) and plasma (**d**) were quantified after 7 days, or at the indicated time points. Expression of the CD11b activation marker on inflammatory Ly6C^high^ blood monocytes was expressed as MFI (**e**). Myeloid cell subsets in infarcted hearts, including macrophages, were quantified after 7 days (**f**, **g**). Rolling and adhering cells in mesenteric venules were quantified in intravital microscopy after depletion of alveolar macrophages and subsequent ROFA exposure (**h**, **i**). Data are presented as mean ± SEM of at least seven mice pre group

Finally, to confirm that alveolar macrophages ultimately determine the inflammatory response in infarcted cardiac tissue after ROFA exposure, we tested a combined model of alveolar macrophage depletion, ROFA exposure, and MI (Fig. 6a). Clodronate depletion effectively reduced alveolar macrophages 7 days after MI (Fig. 6b) and protected from ROFA-mediated increase in BAL and systemic TNF-α levels (Fig. 6c, d). In accord with the finding that blood Ly6C^high^ monocyte activation correlated with plasma TNF-α levels in ROFA-exposed mice (Supplemental Fig. 9), we observed lowered expression of the CD11b activation epitope in circulating inflammatory Ly6C^high^ monocytes in clodronate-pretreated ROFA-exposed mice (Fig. 6e). As a result, lack of alveolar macrophages prevented from macrophage accumulation in infarcted tissue after ROFA exposure (Fig. 6f, g). Moreover, depletion of alveolar macrophages was also effective in reducing the ROFA-mediated increase in leukocyte rolling and adhesion in intravital microscopy (Fig. 6h, i, Supplemental videos IV–VI), suggesting that enhanced inflammatory cell recruitment is the cause of increased macrophage accumulation in infarcted tissue of ROFA-exposed mice.

## Discussion

**Fig. 7:**
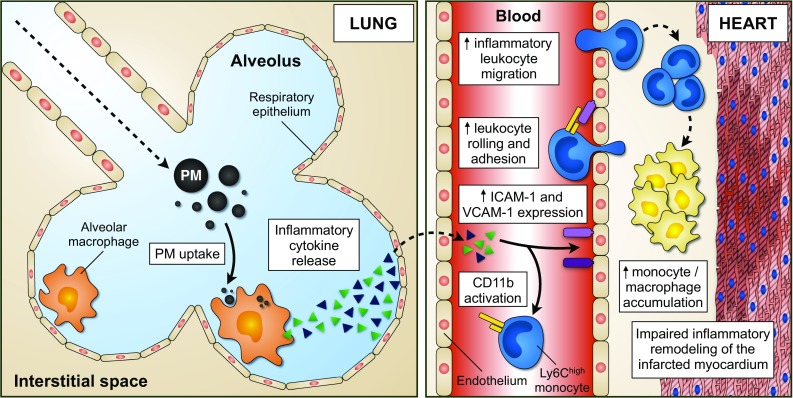
Proposed model of PM-induced aggravation of myeloid cell recruitment during MI. Following PM inhalation, alveolar macrophages are directly activated by PM and orchestrate lung inflammation. The release of pro-inflammatory mediators, such as TNF-α and IL-6, into the blood stream results in increased expression of adhesion factors in myeloid cells and the endothelium. Consequently, leukocyte migration to sites of pre-established inflammation is boosted. Within the infarcted myocardium, monocytes and macrophages accumulate, ultimately leading to impaired tissue remodeling and increased infarction area

Exposure to environmental PM correlates with cardiovascular disease and mortality from MI [1, 3, 5]. However, it is not known whether and by which mechanism PM influence MI. In this study, we present the novel finding that the exposure to ROFA particles aggravates experimental MI by boosting inflammatory cell recruitment to the myocardium. Mechanistically, four major findings support our model (Fig. 7): (1) an acute ROFA exposure induced sustained activation of macrophages, but not of lymphocytes, resident in the lung. (2) Activated alveolar macrophages secreted pro-inflammatory cytokines, such as TNF-α and IL-6, into the circulation. (3) This increase in systemic cytokines promoted expression of myeloid and endothelial adhesion factors, and pre-disposed for enhanced leukocyte rolling, adhesion, and transmigration. Notably, anti-cytokine therapy reversed these effects. (4) Experimental depletion of alveolar macrophages reduced inflammatory cytokine release and protected from inflammatory cell recruitment induced by ROFA in vivo. Accordingly, attenuation of myeloid cell recruitment into the myocardium has been reported to improve MI healing [31].

Numerous studies have previously suggested different mechanisms. Firstly, a direct uptake of PM by phagocytes, such as by macrophages, has been demonstrated [21]. Secondly, it is thought that certain PM constituents may induce an oxidative stress response [50]. Airborne PM is comprised of a heterogeneous mixture of solid and liquid particles suspended in air, varying in size, chemical composition and sources of origin [5]. Anthropogenic emissions are the main contributors to environmental PM burden and mainly consist of motor vehicle emissions and fossil fuel combustion during power generation and industrial processes [32]. ROFA particles often present an aerodynamic diameter below 2.5 μm (PM_2.5_), a size that penetrates the lung deeper and has been shown to be more closely associated with PM adverse health effects rather than coarser particles (PM_10_) [5]. ROFA particles are especially rich in soluble transition metals, that can enhance the inflammatory response triggered by PM, through generation of reactive oxygen species (ROS) [7]. In our study, we tested PM rich in water-soluble transition metals [8, 36, 38]. Some deleterious effects observed in local lung injury appear to be caused by reactive oxygen species which may potentially be overcome by over-expression of superoxide dismutase [17, 18, 27, 28]. An interesting link is provided by the observation that increased oxidative damage after PM exposure leads to the generation of oxidized lipid species, such as 1-palmitoyl-2-arachidonoyl-*sn*-glycero-3-phosphorylcholine (oxPAPC) in the bronchoalveolar lavage fluid. The later can be engulfed by macrophages mediated by pattern-recognition receptors, such as Toll-like receptors (TLRs), and potentiate inflammatory signaling [24]. In accord, TLR4-deficient mice are protected from PM-associated inflammation [24] and MI [37]. In our study, TLR4 expression was enhanced in alveolar macrophages after ROFA exposure, but not in circulating leukocytes, raising the possibility that TLR4 may indeed mediate some inflammatory pathways initiated by ROFA. However, we also observed that ROFA particles alone induced pro-inflammatory cytokine expression in isolated alveolar macrophages, indicating that such pathways are not required for, but rather synergistically support the inflammatory response after ROFA exposure.

The concept that PM exposure induces leukocyte recruitment by promoting both rolling and firm adhesion has been reported earlier. In particular, Nurkiewicz et al. have shown that rats exposed to ROFA exhibited increased leukocyte deposition in the spinotrapezius muscle [34]. This effect was discussed to be caused by deposition of myeloperoxidase in the arterial wall [35]. Moreover, we previously showed that ROFA instillation activates intravascular neutrophils [28], as also confirmed in this study. However, it has not been elucidated whether leukocytes are activated per se or whether this activation is secondary to circulating mediators of inflammation. By testing conditioned plasma from ROFA-exposed mice on in vitro cultured leukocytes and endothelial cells, we showed that cytokines are needed to induce cell activation, while ROFA particles alone are not sufficient to fully activate cell types participating in leukocyte recruitment. In vitro, circulating TNF-α levels in ROFA-exposed mice caused ICAM-1 and VCAM-1 expression in endothelial cells, as well as CD11b (Mac-1) integrin activation in myeloid cells, a prerequisite for leukocyte adhesion to ICAM-1 [9, 13]. This is particularly interesting since TNF-α is capable of inducing endothelial activation [23]. Given that nano-scale particles and soluble PM constituents have been found in systemic circulation after PM inhalation [33, 45], a slight effect of ROFA particles on endothelial and circulating leukocytes cannot be completely ruled out. As proof-of-concept, we have demonstrated that therapeutic inhibition of the prototypic pro-inflammatory cytokine TNF-α reverses the effects of PM. In accord, it has been recently shown that TNF-α blockade is also effective to prevent PM-associated worsening in cardiac energetic and contractile function [30]. Our findings do not exclude the participation of alternative pro-inflammatory mediators. In particular, Fijimaki et al. have demonstrated that IL-6-deficient mice failed to accumulate leukocytes in the lung after the exposure to diesel exhaust particles [12], proposing that IL-6 also participates in lung inflammation in this setting.

In our study, alveolar macrophages are the source of TNF-α and IL-6 after ROFA exposure. These data confirm previous studies showing that macrophage activation occurs in vitro and in vivo after exposure to PM [21]. It has already been demonstrated that cytokine levels detectable in BAL rise after PM inhalation [11, 17]. However, the cellular origin of these cytokines has not been tracked down until now. Another important finding from our study is that circulating TNF-α levels rise following PM exposure, even in the context of already elevated TNF-α plasma levels after MI. Notably, lymphocytes can promote acute and chronic inflammation by cytokine secretion and providing chemotactic stimuli for myeloid cells, particularly in the setting of MI [55]. To further elucidate the relative participation of lymphocytes and macrophages, Rag1^−/−^ mice lacking mature lymphocytes and clodronate-pretreated mice lacking alveolar macrophages were used as experimental models. Interestingly, local and systemic inflammation in Rag1^−/−^ mice resembled that of wild type mice, indicating that T- and B-lymphocytes do not play a relevant role in this context. On the contrary, we observed that some cytokine levels were even higher in Rag1^−/−^ than in wild type mice, suggesting a protective role of some lymphocyte subsets, as previously suggested for T-regulatory cells in lung inflammation [54]. Specific depletion of alveolar macrophages reduced local and systemic inflammation after ROFA exposure, indicating that alveolar macrophages are the source of circulating pro-inflammatory cytokines in our model. Of note, we have not observed that i.n. clodronate affects myeloid cell survival at other locations than the lung. This is particularly important, since MI is driven by the mobilization of monocytes from secondary lymphoid organs and the bone marrow to the heart, where they rapidly differentiate into macrophages within a couple of days [31]. Accordingly, lack of alveolar macrophages prevented excessive inflammatory leukocyte accumulation in infarcted tissue of ROFA-exposed mice. In this context, it is also possible that ROFA exposure may trigger the release of monocytes from the bone marrow of lymphoid organs, although we have not observed elevated numbers of circulating myeloid cells following ROFA exposure. On the other hand, other effector functions of alveolar macrophages apart from cytokine release may also contribute to the observed effects. These questions will have to be answered in future studies.

Despite ROFA is an accepted model particle for air pollution PM [16], a potential limitation of our study is that an acute exposure to ROFA may only partially reflect environmental exposures as present in urban areas of industrialized countries, in particular when PM is present at lower concentrations. However, acute exposure to oil fly ashes [15, 19, 25] in humans has been reported to induce a comparable degree of lung injury and inflammation as observed in the present study.

In conclusion, the presented findings unravel some of the key mechanisms by which PM worsens the outcome following MI, directly linking air pollution PM with the activation of pathologic inflammatory pathways. After all, our data highlight the importance of environmental factors in cardiovascular disease.

## Electronic supplementary material

Below is the link to the electronic supplementary material.

Supplementary material 1 (PDF 1453 kb)

Supplementary material 2 (MOV 383 kb)

Supplementary material 3 (MOV 381 kb)

Supplementary material 4 (MOV 379 kb)

Supplementary material 5 (MOV 1214 kb)

Supplementary material 6 (MOV 1219 kb)

Supplementary material 7 (MOV 1229 kb)
