# Supplementary material for: Acute exposure to air pollution particulate matter aggravates experimental myocardial infarction in mice by potentiating cytokine secretion from lung macrophages
Source: Basic Res Cardiol. 2016 May 30;111:44. doi: 10.1007/s00395-016-0562-5 (PMC4886146; doi:10.1007/s00395-016-0562-5)
Supplement: Supplementary file 1 — Supplementary material 1 (PDF 1453 kb) [file 395_2016_562_MOESM1_ESM.pdf]

ONLINE DATA SUPPLEMENT

"Acute exposure to air pollution particulate matter aggravates experimental myocardial infarction in mice by potentiating cytokine secretion from lung macrophages"

EXPANDED METHODS SECTION

Particulate matter (PM). ROFA particles were collected from Boston Edison Co., Mystic Power Plant, Mystic, CT, US burning low-sulfur residual oil (No. 6 fuel oil) and were kindly provided by Dr. J. Godleski (Harvard School of Public Health, Harvard University, Boston, MA, US). ROFA samples from this source have been extensively characterized for elemental composition and particle size. Vanadium, nickel, and iron are the predominant metals present as water-soluble sulfates and particle mean aerodynamic diameter is 2.06 ± 1.57 µm [1, 4, 7, 8]. PM samples were freshly prepared by suspending ROFA particles in sterile saline solution at 5 mg/ml, followed by incubation in an ultrasonic water bath for 5 min before use.

In vivo exposure to PM. Male 8-week-old wild type or Rag1-deficient ($Rag1^{-/-}$) mice on a C57BL/6J background were used as indicated (Jackson Laboratories, Bar Harbor, ME, US). Animals were anesthetized by isoflurane and exposed to ROFA particles (1 mg/kg body weight) or saline solution (control group) by intranasal (i.n.) instillation. Mice were immobilized in a 60° inclined supine position while 50 µl of the ROFA suspension was delivered dropwise to the nares by the use of an automatic pipette. Control mice were handled in parallel and instilled with 50 µl of sterile saline solution (vehicle). Due to the presence of fluid in the mouse nasal cavity, a respiratory reflex is triggered which ensures that the maximum delivered volume reaches the lung [9]. The selected dose falls within the range of concentrations consistently used in several animal studies [2, 5, 6]. All experimental protocols were approved by the animal ethics committee of the University of Freiburg,

Freiburg, Germany, and every procedure was carried out in accordance with institutional guidelines.

Murine model of myocardial infarction (MI). MI was induced to C57BL/6J mice by permanent ligation of the left anterior descending coronary artery (LAD) as described earlier [3]. Mice were anesthetized with isoflurane (2%/2 litters O_2), immobilized on a heating pad, intubated, and mechanically ventilated with a rodent respirator. Left thoracotomy was performed in the fourth intercostal space after shaving the chest wall. The left ventricle was visualized and the LAD was ligated with monofilament 8-0 suture (Ethicon, Somerville, NJ, US). Pallor of the perfusion bed ensured myocardial ischemia. The chest and skin were closed with a 7-0 nylon suture (Ethicon) after removal of air from the thorax via a pleural catheter. Mice were exposed to sterile saline solution or ROFA particles on a daily basis, starting one day before the surgery and up to one week.

Histology. On day 7 after MI, hearts were embedded in Tissue-Tek O.C.T. compound (Sakura Finetek, Torrance, CA, US), frozen in ice-cold 2-Methylbutane (Fisher Scientific, Fair Lawn, NJ, US), and sectioned into 6 μm slices. Infarct area was demarked in cross sections of paraffin-embedded hearts by Masson's Trichrome staining. To evaluate myeloid cell infiltration, immunohistochemical staining for CD11b was performed using an anti-CD11b antibody clone M1/70 (BD Biosciences, San Jose, CA, US) and counterstained with hematoxylin. Images were recorded using an AxioPlan 2 microscope (Carl Zeiss, Oberkochen, Germany) and analyzed with ImagePro (Media Cybernetics, Rockville, MD, US). Extent of leukocyte infiltration was expressed as $CD11b^+$ area (%) of the total infarcted tissue.

Electrocardiography (ECG). Electrodes were implanted subcutaneously in a Lead II configuration (right arm, left leg, and right leg) in mice under isoflurane anesthesia. Data were acquired after the ROFA exposure on days 0, 1, 3, and 7 after LAD ligation in a 701

EGM module (Harvard Apparatus, March-Hugstetten, Germany) equipped with a PowerLab 8/35 data acquisition system (ADInstruments, Oxford, United Kingdom). ECG data was analyzed with LabChart Pro (ADInstruments).

Intravital microscopy. C57BL/6J mice were anesthetized with an i.p. injection of ketamine (185 mg/kg body weight) and xylazine (60 mg/kg body weight). Platelets and white blood cells were fluorescently labeled by a retro-orbital injection of 60 µl Rhodamine 3G (1 mg/ml). Mice were kept on a heating pad to maintain body temperature and intravital microscopy was performed as previously described [11]. A loop of ileum was exteriorized by a longitudinal abdominal skin incision and subsequent dissection of facial and peritoneal layers. A small mesenteric vein with a diameter of approximately 100 µm was chosen for analysis. Videos were taken with an intravital microscope (AxioScope Vario, Carl Zeiss, Germany) fitted with a saline immersion objective (WPlan-APOCHROMAT 20x/1,0DIC IR, Carl Zeiss, Germany) and a high sensitivity camera system (AxioCam MRm, Carl Zeiss, Germany) for 30 seconds. Rolling leukocyte flux was defined as the number of leukocytes moving at a lower velocity than erythrocytes. Rolling velocity was calculated from the average time needed for one single leukocyte to roll over the endothelium for a distance of 100 µm. Adherent leukocytes were defined as cells that remained stationary for at least 30 seconds. Measurements were performed 3 hours after the acute ROFA exposure. As positive control, non-exposed mice received an i.p. injection of 200 ng murine TNF-α (R&D Systems, Minneapolis, MN, US) 4 hours prior to imaging. Rolling flux, adhering leukocytes, and rolling velocity were quantified by blinded investigators.

Blood samples. Blood samples were collected 3 hours after the ROFA exposure by retro-orbital bleeding with EDTA as anticoagulant. Plasma was separated by centrifugation at 600 g for 5 min at 4 °C, and kept at -20 °C until further use. The leukocyte pellet was used for flow cytometry. Blood neutrophils were identified as $CD45^+$, $CD11b^+$, $CD115^-$, $Ly6C^{int}$, $Gr\text{-}1^+$ cells. Monocytes were identified as $CD45^+$, $CD11b^+$, $CD115^+$ cells, and subdivided into

Ly6C^{high} (CD45$^+$, CD11b$^+$, CD115$^+$, Ly6C^{high}, Gr-1$^+$) and Ly6C^{low} (CD45$^+$, CD11b$^+$, CD115$^+$, Ly6C^{low}, Gr-1$^-$) subsets. T-cells were identified as CD45$^+$, CD3$^+$ cells, B-cells as CD45$^+$, CD19$^+$ cells.

Flow cytometry. Cell suspensions were analyzed by flow cytometry in a FACS Canto II cytometer (BD Bioscience, Franklin Lakes, NJ, US) as previously described [3, 11]. Briefly, red blood cells were lyzed in 155 mM NH_4Cl, 5.7 mM K_2HPO_4, 0.1 mM EDTA, pH 7.3. Leukocytes were resuspended in 0.1% BSA/PBS and Fc-receptors were blocked with anti-CD16/CD32 antibodies (clone 93, eBioscience, San Diego, CA, US) for 10 min on ice. Samples were stained as indicated in 0.1% BSA/PBS during 25 min on ice in the dark. For intracellular cytokines quantification, cells were fixed and permeabilized using the Cytofix/Cytoperm kit (BD Biosciences) according to manufacturer's instructions and incubated with conjugated anti-TNF-α, anti-IL-6, and anti-MCP-1 antibodies. Antibodies for epitope specific fluorescence-activated cell sorting included: anti-CD3 (clone 145-2C11, eBioscience), anti-CD11b (clones M1/70 and CBRM1/5, eBioscience), anti-CD11c (clone N418, eBioscience), anti-CD19 (clone 1D3, eBioscience), anti-CD41 (clone MWReg30, eBioscience), anti-CD45.2 (clone 104, eBioscience), anti-CD49b (clone DX5, eBioscience), anti-CD54/ICAM-1 (clone KAT-1, eBioscience), anti-CD62E/E-Selectin (clone 10E9.6, BD Bioscience), anti-CD62P/P-Selectin (clone KO2.3, eBioscience), anti-CD64 (clone X54-5/7.1, BioLegend), anti-CD68 (FA-11, BioLegend), anti-CD106/VCAM-1 (clone 429, BioLegend), anti-CD115 (clone AFS98, eBioscience), anti-CD284/TLR4/MD-2 (clone MTS510, BioLegend), anti-F4/80 (clone BM8, BioLegend), anti-IL-6 (clone MP5-20F3, eBioscience), anti-Ly6C (clone HK1.4, BioLegend), anti-Ly6G/Gr-1 (clone RB6-8C5, eBioscience), anti-MCP-1 (clone 2H5, eBioscience), anti-MHCII (clone AF6-120.1, BioLegend), anti-Siglec-F (clone E50-2440, BD Bioscience), anti-TNF-α (clone MP6-XT22, eBioscience), and isotype controls. Leukocyte populations resident in the heart were quantified in digested samples on days 0, 3, and 7 after LAD ligation by flow cytometry as previously described [3]. Briefly, hearts were excised and infarcted tissue was dissected, weighted, and incubated in digestion

mix (450 U/ml collagenase I, 125 U/ml collagenase IX, 60 U/ml hyaluronidase, 60 U/ml DNAse I, 5 mM $CaCl_2$, 20 mM HEPES) in PBS for 45 min at 37 °C with continuous shaking at 750 rpm. After being washed and filtered through a 70 μm nylon mesh, cells were counted in a hemocytometer and stained for flow cytometry. Cardiac monocytes were identified as $CD45^+$, Lin^- (Lin = CD3, CD19, CD49b, Ly6G, NK1.1), $CD11b^+$, $F4/80^{low}$, $MHCII^{low}$, $CD11c^{low}$, $CD68^{low}$, $CD115^+$ cells, subdivided into $Ly6C^{high}$ and $Ly6C^{low}$ subsets; neutrophils as $CD45^+$, Lin^+, $CD11b^+$, $MHCII^{low}$, $CD11c^{low}$, SSC^{high}, $Ly6C^{int}$ cells; and cardiac macrophages as $CD45^+$, Lin^-, $CD11b^+$, $F4/80^{high}$ ($MHCII^+$, $CD68^{high}$) cells. Data was analyzed with FlowJo (Tree Star, Ashland, OR, US).

Primary mouse endothelial cells (mEC). mEC were isolated from non-exposed C57BL/6J mice as previously described [11]. Briefly, animals were euthanized with CO_2 and lungs, heart, brain, spleen, and liver were harvested in a sterile environment. Tissue was minced with a razor blade and digested in 0.2% collagenase I (Worthington, Lakewood, NJ, US) in 1% BSA/PBS for 60 min at 37 °C. After being washed and filtered through a 70 μm nylon mesh, cells were resuspended in 0.1% BSA/PBS and incubated for 10 min at room temperature with an anti-mouse CD31/PECAM-1 antibody (clone MEC 13.3, BD Biosciences) conjugated with sheep anti-rat Dynabeads (Dynal Biotech, Oslo, Norway). Cells were then separated and washed using a magnetic particle concentrator (Dynal Biotech), and seeded into gelatin-coated T25 plates. After reaching confluence, a second magnetic sorting was performed with an anti-mouse CD102/ICAM-2 antibody (clone 3C4, BD Biosciences). After being separated and washed, isolated endothelial cells were seeded into gelatin-coated T75 plates. Cells were grown in DMEM high glucose supplemented with 20% fetal bovine serum, 1% sodium pyruvate, 1% heparin, 1% bovine endothelial growth factor, 0.6% non-essential amino acids, and 1% penicillin/streptomycin.

In vitro cell activation assays. mEC were detached from T75 plates using Accutase (Life Technologies, Carlsbad, CA, US), washed with 0.1% BSA/PBS, and seeded into 48-weel

plates at 1x10^5 cells/ml DMEM. Plasma (1% v/v) from saline- or ROFA-exposed mice, or ROFA particles (1 µg/ml) was added as indicated. To evaluate the effects of plasma TNF-α, IL-6, and MCP-1 on mEC activation, plasma samples were pre-incubated on ice for 10 min with a blocking anti-TNF-α, anti-IL-6, or anti-MCP-1 antibody (BD Biosciences) at 10 µg/ml. After 24 hours at 37 °C, mEC culture supernatants were frozen and kept at -20 °C until analyzed for cytokine levels by the CBA assay. Cells were washed with 0.1% BSA/PBS and detached using Accutase (Life Technologies). Endothelial expression of adhesion molecules (CD54/ICAM-1, CD106/VCAM-1, CD62P/P-Selectin, and CD62E/E-Selectin) was analyzed by flow cytometry.

Immunohistochemistry. mEC were detached from T75 plates using Accutase (Life Technologies), washed with 0.1% BSA/PBS, and seeded into cell culture slides (BD Biosciences). When confluence was reached, ROFA particles (1 µg/ml) were added. TNF-α (10 ng/ml) (R&D Systems) was used as a positive control. After 24 hours at 37 °C, slides were washed with PBS, fixed with acetone, and permeabilized with 0.1% Triton X-100/PBS. Nonspecific binding was blocked with a 5% species-appropriate normal serum (Vector Laboratories, Burlingame, CA, US). Sections were then incubated with primary rabbit anti-mouse ICAM-1 or goat-anti mouse VCAM-1 antibodies (Santa Cruz Biotechnology, Heidelberg, Germany). After 1 hour at room temperature in the dark, slides were washed with PBS and incubated with a goat anti-rabbit Alexa Fluor647 antibody for ICAM-1 staining, and with a rabbit anti-goat FICT antibody for VCAM-1 staining (Life Technologies). After incubation with the secondary antibody, slides were washed, counterstained with a 1:30000 4',6-diamidino-2-phenylindole (DAPI) dilution, and embedded with fluorescence mounting medium (Carl Roth GmbH, Karlsruhe, Germany). Staining with the corresponding IgG isotype antibodies (BD Biosciences) served as control.

Leukocyte isolation. EasySep (Stem Cell Technologies, Vancouver, Canada) cell enrichment kits were used according to manufacturer's protocols. Neutrophils were isolated by negative

selection from peritoneal lavage fluid, obtained 4 hours after the induction of sterile peritonitis by an i.p. injection of 2 ml 4% thioglycollate broth (Sigma). Monocytes and macrophages were isolated by negative selection from a splenocyte cell suspension, obtained from the spleen by nicking the capsule and gently rotating two microscope slides. The obtained cell suspension was filtered through a 100 µm nylon mesh, pelleted, and used for cell isolation. Cell purity was >98% in every leukocyte subtype preparation as assessed by flow cytometry.

In vitro cell activation assays. As indicated, isolated leukocytes were seeded into 96-weel plates at $1x10^5$ cells/ml RPMI media, and incubated with plasma (1% v/v) from saline- or ROFA-exposed mice, or ROFA particles (1 µg/ml). To evaluate the contribution of plasma TNF-α, IL-6, and MCP-1 on leukocyte activation, plasma samples were pre-incubated on ice for 10 min with a blocking anti-TNF-α, anti-IL-6, or anti-MCP-1 antibody (BD Biosciences) at 10 µg/ml. After 24 hours at 37 °C, cell culture supernatants were frozen and kept at -20 °C until analyzed for cytokine levels by the CBA assay. Leukocytes were washed with 0.1% BSA/PBS, detached using Accutase (Life Technologies), and analyzed by flow cytometry.

Bronchoalveolar lavage (BAL). Mice were euthanized with CO_2 3 hours after the ROFA exposure and a BAL was performed as previously described. Briefly, the trachea was cannulated and lungs were flushed 3 times with 1 ml 0.1% BSA/PBS each. BAL fluid total cells were counted in a hemocytometer. Samples were centrifuged at 600 g for 5 min at 4 °C and BAL fluid supernatant was stored at -20 °C until assayed for cytokine levels by the CBA assay. The obtained cell pellet was assayed for leukocyte subsets, TLR4 expression, and intracellular cytokines by flow cytometry. Alveolar macrophages were identified as $CD45^+$, $CD11b^{low}$, Siglec-F^+, $CD11c^+$, $CD64^+$, $F4/80^{int}$ cells (Supplemental Fig. 2).

Macrophage depletion. Alveolar macrophages were depleted from C57BL/6J mice before ROFA exposure by an i.n. instillation of 50 µl dichloromethylene bisphosphonate (clodronate) liposomes (5 mg/mL) (VU University Medical Center, Amsterdam, The Netherlands) as

previously described [12]. Alternatively, 200 µl clodronate liposomes were injected i.v. for systemic depletion of macrophages before ROFA exposure. PBS-loaded liposomes were used as control. Clodronate i.n. resulted in a selective depletion of alveolar macrophages by ~90% after 24 hours (Supplemental Fig. 5a) while other leukocyte populations, such as myeloid cells in blood and spleen, were not significantly affected (Supplemental Fig. 5b, c). Alveolar macrophage depletion in ROFA-exposed mice lasts for up to 5 days after a single i.n. clodronate dose (Supplemental Fig. 6). As alveolar macrophages start to significantly repopulating the lung within days 3 and 4 after i.n. clodronate delivery, mice received i.n. clodronate every third day in the combined model of alveolar macrophage depletion, ROFA exposure, and MI.

Statistics. Data are presented as mean ± SEM. Unpaired Student's t-test was used to analyze differences between two groups. ANOVA followed by the Student–Newman–Keuls *post-hoc* test was performed to evaluate differences between more than two groups. Statistical significance was considered at $p<0.05$.

Supplementary Table 1: ECG characteristics after induction of MI.

	Day 0		Day 1		Day 3		Day 7	
	**Saline**	**ROFA**	**Saline**	**ROFA**	**Saline**	**ROFA**	**Saline**	**ROFA**
**1/R-R (1/ms x10^{-3})**	3.7±0.2	4.0±0.2	8.0±0.3	8.0±0.3	8.0±0.3	8.7±0.3	8.7±0.5	8.7±0.2
**QRS (ms)**	9.5±0.3	10.7±0.5*	5.7±0.3	6.7±0.7	6.0±0.4	8.3±0.8*	7.3±0.9	7.3±0.6
**PQ (ms)**	33.2±0.9	33.3±1.9	34.3±1.0	35.0±0.5	36.6±0.9	33.3±0.9	35.0±0.7	34.5±0.7
**QT (ms)**	32.5±1.4	33.1±1.4	28.3±2.9	27.5±2.9	31.7±1.6	32.0±2.1	34.0±0.2	32.3±2.0
**Heart freq. (bpm)**	226±11	241±7	482±19	530±16	481±16	522±19	522±27	524±12

*p<0.05 compared to the saline group at the same time point. ms indicates milliseconds, bpm indicates beat per minute.

SUPPLEMENTARY FIGURES AND FIGURES LEGENDS

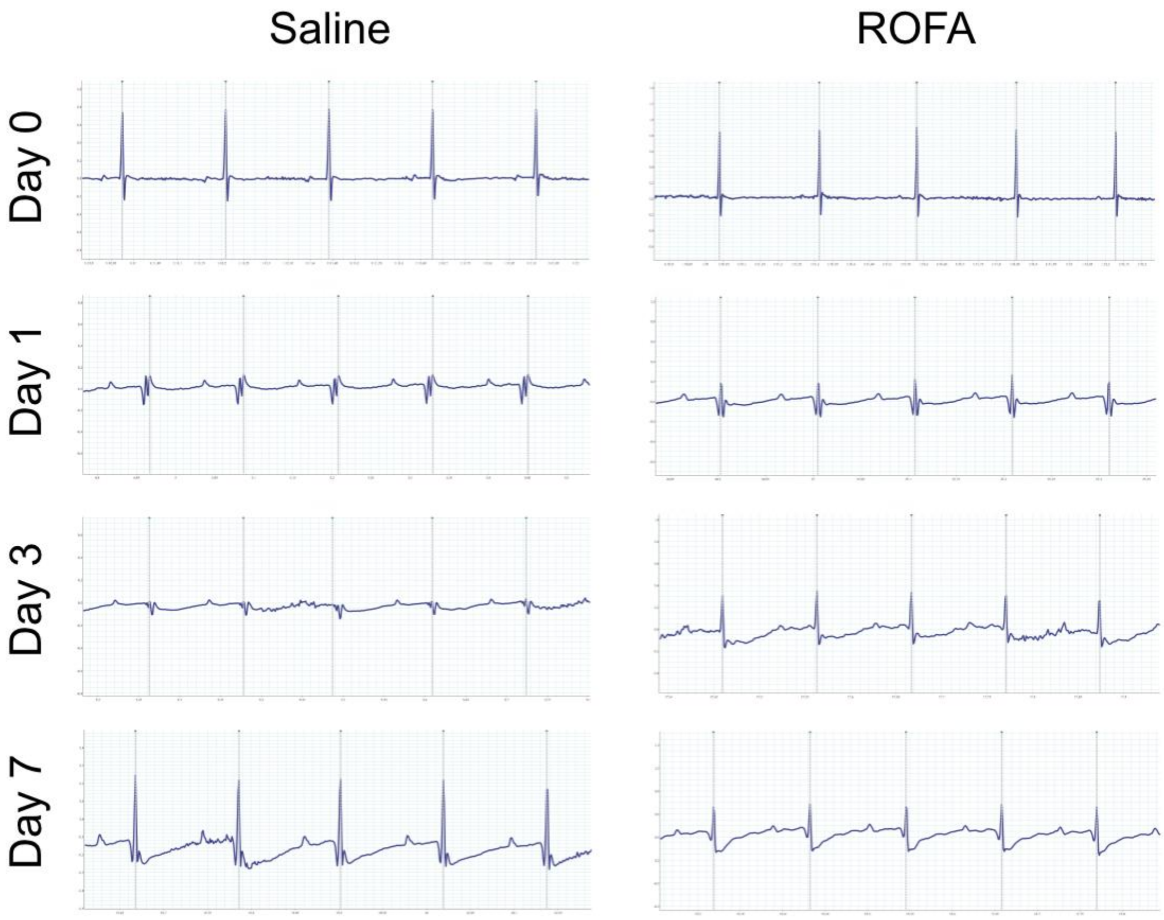

Supplementary Fig. 1: Representative ECG of saline- and ROFA-treated mice after surgical induction of MI. Data were acquired after ROFA or saline exposure on days 0, 1, 3, and 7 after LAD ligation in a 701 EGM module. ECG showed the typical ST-changes after MI, but did not lead to an increased rate of arrhythmias or premature ventricular complexes at the indicated time points in both groups.

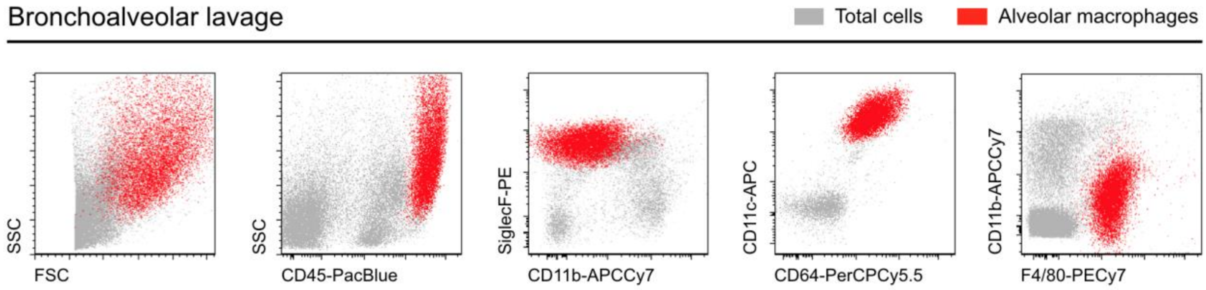

Supplementary Fig. 2: Gating strategy for flow cytometry of alveolar macrophages. Surface marker expression profile of alveolar macrophages from BAL samples of untreated C57BL/6J mice. Alveolar macrophages were identified as $CD45^{+}$, $CD11b^{low}$, Siglec-F^{+}, $CD11c^{+}$, $CD64^{+}$, $F4/80^{int}$ cells and depicted in red in the backgating. Total BAL cells are depicted in grey.

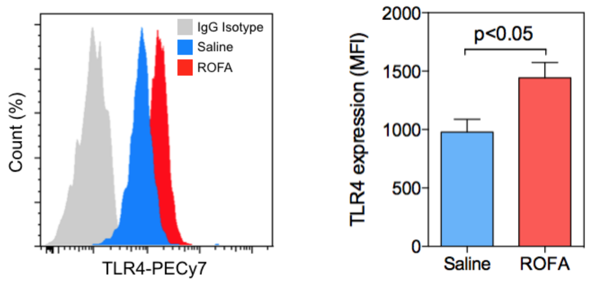

Supplementary Fig. 3: Exposure to ROFA induces expression of Toll-like Receptor (TLR) 4 in alveolar macrophages. C57BL/6J mice were exposed to saline solution or ROFA particles (1 mg/kg body weight) by i.n. instillation. A BAL was performed after 3 hours and TLR4 expression was quantified in alveolar macrophages by flow cytometry (expressed as mean fluorescence intensity, MFI). Data are presented as mean ± SEM of at least 6 mice per group.

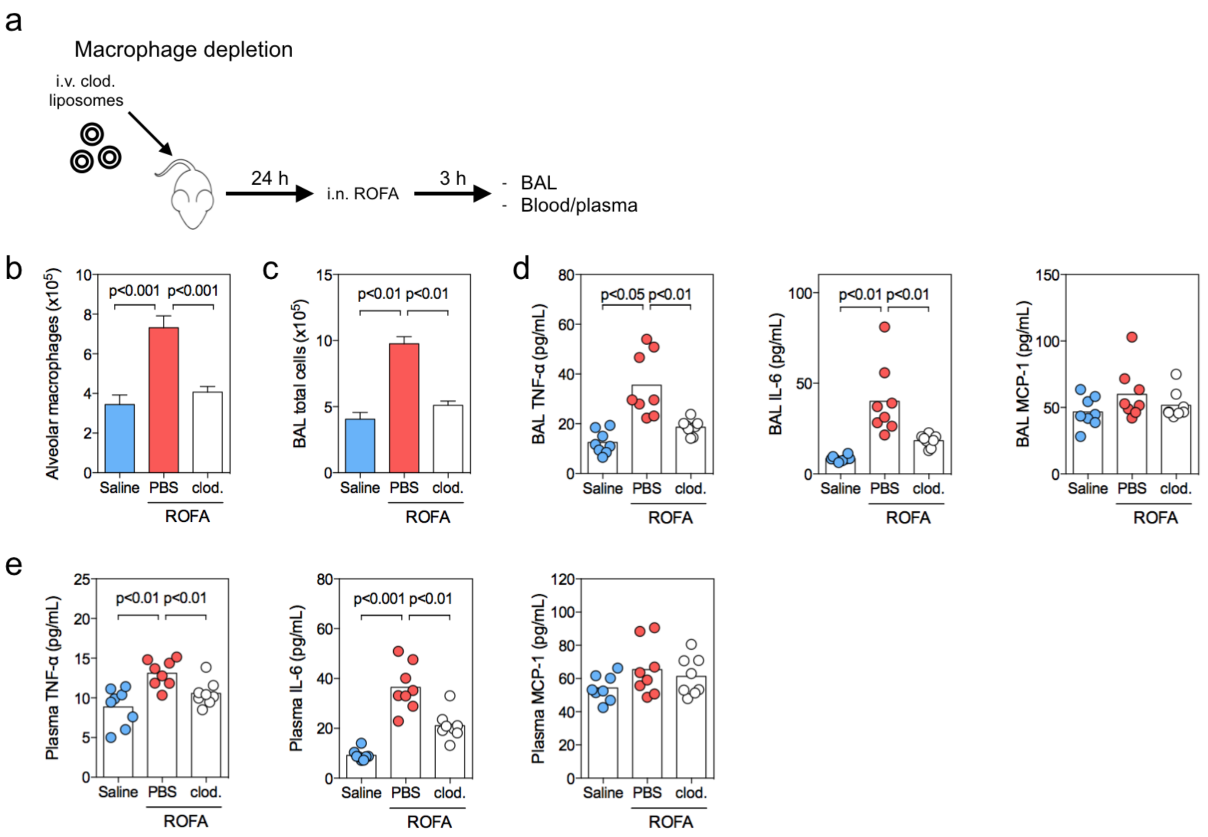

Supplementary Fig. 4: Systemic depletion of macrophages protects from ROFA-mediated cytokine release. Liposomes containing clodronate or PBS were injected i.v. to systemically deplete macrophages from C57BL/6J mice. 24 hours later, mice were exposed to ROFA (1 mg/kg body weight) by i.n. instillation and sacrificed 3 hours after the treatment (a). BAL cells were counted or analyzed by flow cytometry (b, c). Levels of pro-inflammatory cytokines in BAL (d) or plasma (e) were quantified by a cytometric bead array. Data are presented as mean ± SEM of at least 8 mice per group. PBS indicates PBS-containing liposomes, clod. indicates clodronate-containing liposomes. P-values > 0.05 are not indicated.

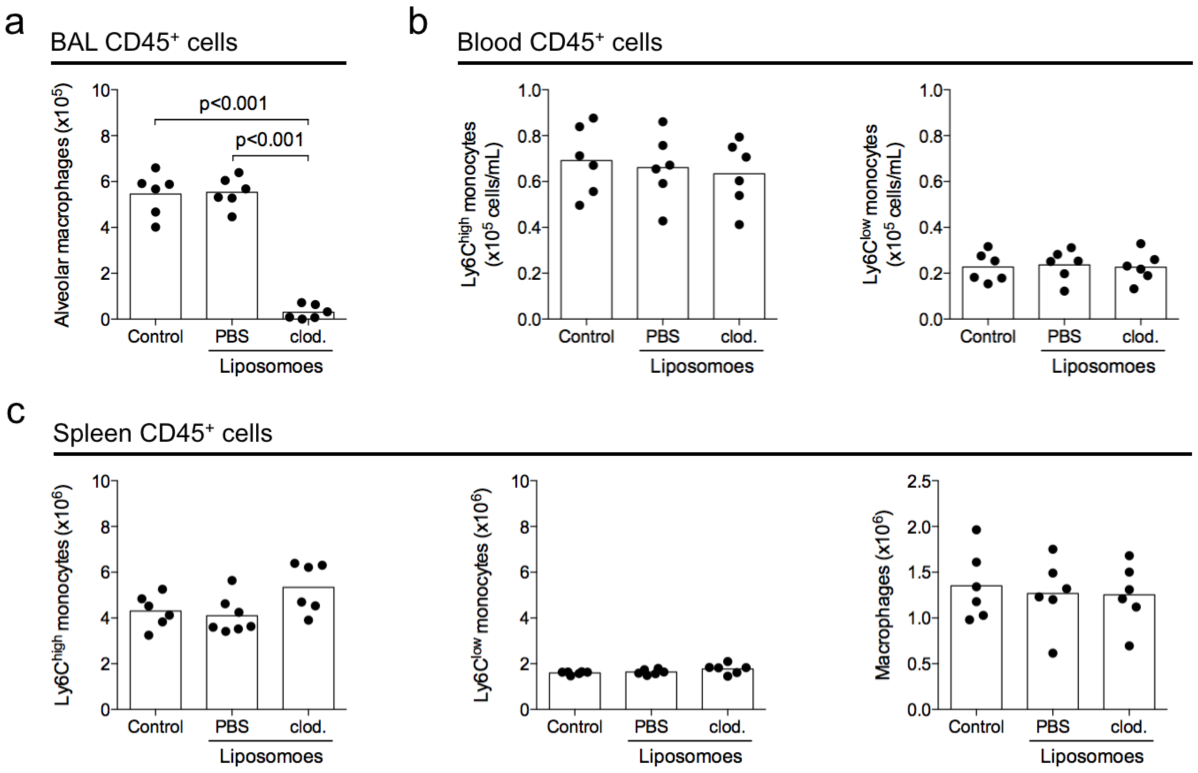

Supplementary Fig. 5: Intranasal instillation with clodronate liposomes is specific for alveolar macrophage depletion. Untreated C57BL/6J mice received 50 μl clodronate liposomes by i.n. instillation. PBS-loaded liposomes were used as control. After 24 hours, myeloid cell populations were profiled in BAL (a), blood (b), and spleen (c). Data are presented as mean ± SEM of 6 mice per group. PBS indicates PBS-containing liposomes, clod. indicates clodronate-containing liposomes. P-values > 0.05 are not indicated.

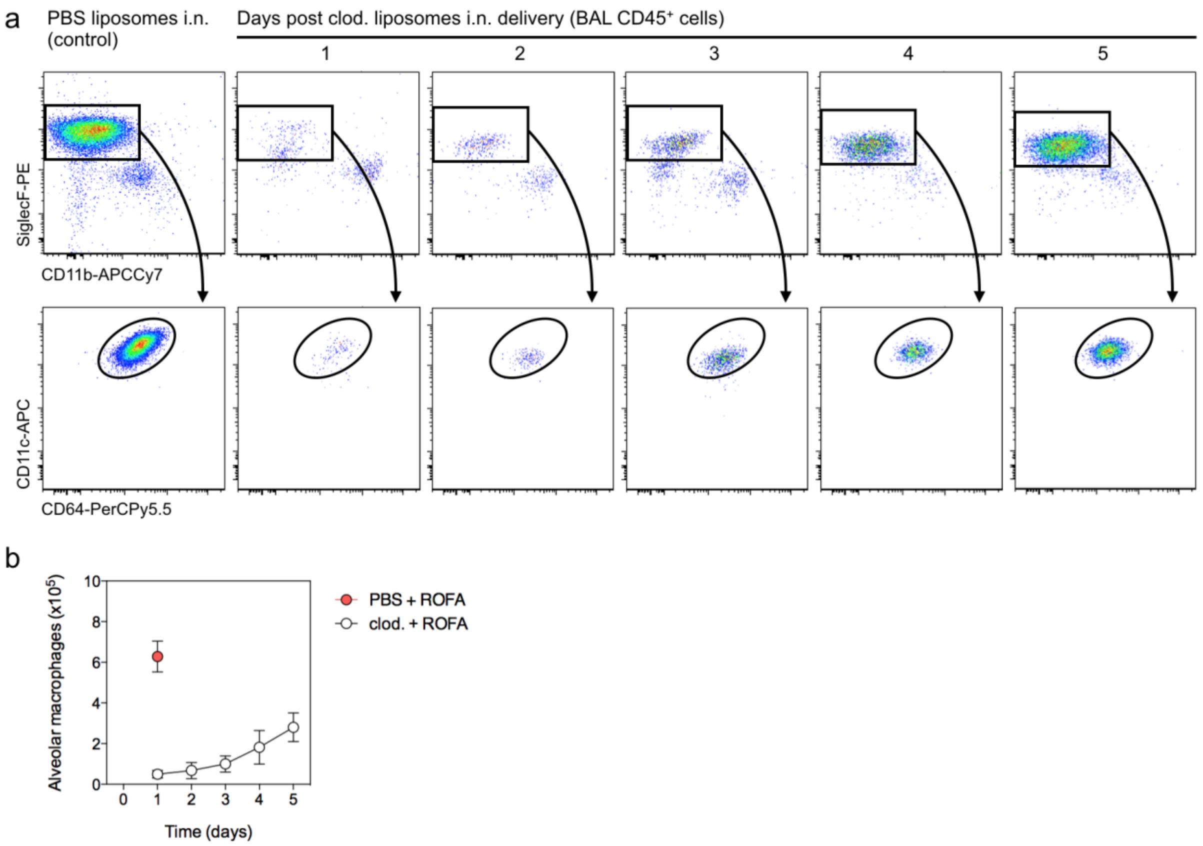

Supplementary Fig. 6: Kinetics of alveolar macrophage depletion following i.n. clodronate in ROFA-exposed mice. C57BL/6J mice received clodronate liposomes by i.n. instillation. After 24 hours, mice were exposed to ROFA (1 mg/kg body weight) on a daily basis and BAL alveolar macrophages were profiled by flow cytometry (a, b). ROFA-exposed mice pretreated with PBS-loaded liposomes were used as a control. Data are presented as mean ± SEM of at least 3 mice per group. PBS indicates PBS-containing liposomes, clod. indicates clodronate-containing liposomes.

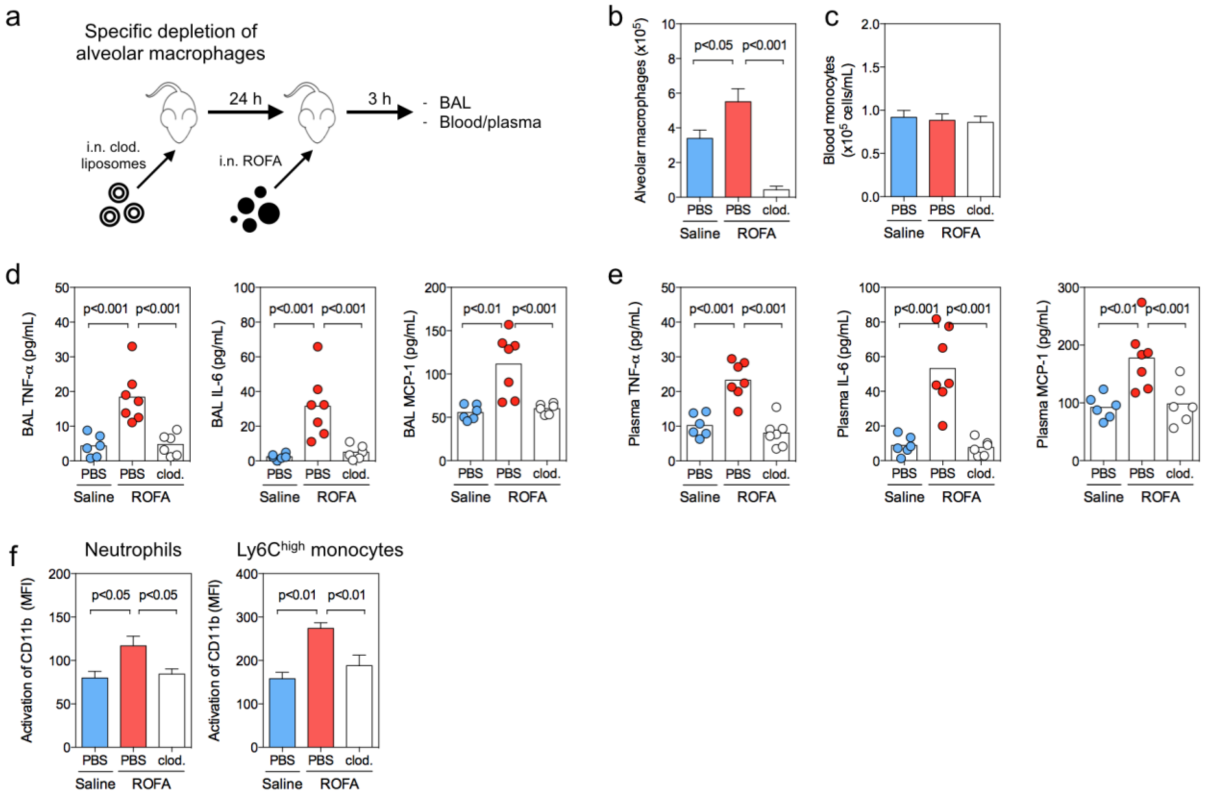

Supplementary Fig. 7: Specific depletion of alveolar macrophages prevents ROFA-mediated local and systemic cytokine release. Alveolar macrophages were depleted in C57BL/6J mice by an i.n. delivery of clodronate liposomes. PBS-loaded liposomes were used as control. 24 hours later, animals were exposed to ROFA (1 mg/kg body weight) and sacrificed 3 hours after the treatment (a). BAL alveolar macrophage numbers (b) and total blood monocytes (c) were evaluated by flow cytometry. Levels of pro-inflammatory cytokines in BAL (d) or plasma (e) were quantified by a cytometric bead array. Expression of the CD11b activation marker CBRM1/5 on blood neutrophils and inflammatory Ly6C^{high} monocytes was expressed as mean fluorescence intensity (MFI) (f). Data are presented as mean ± SEM of at least 8 mice per group. PBS indicates PBS-containing liposomes, clod. indicates clodronate-containing liposomes. P-values > 0.05 are not indicated.

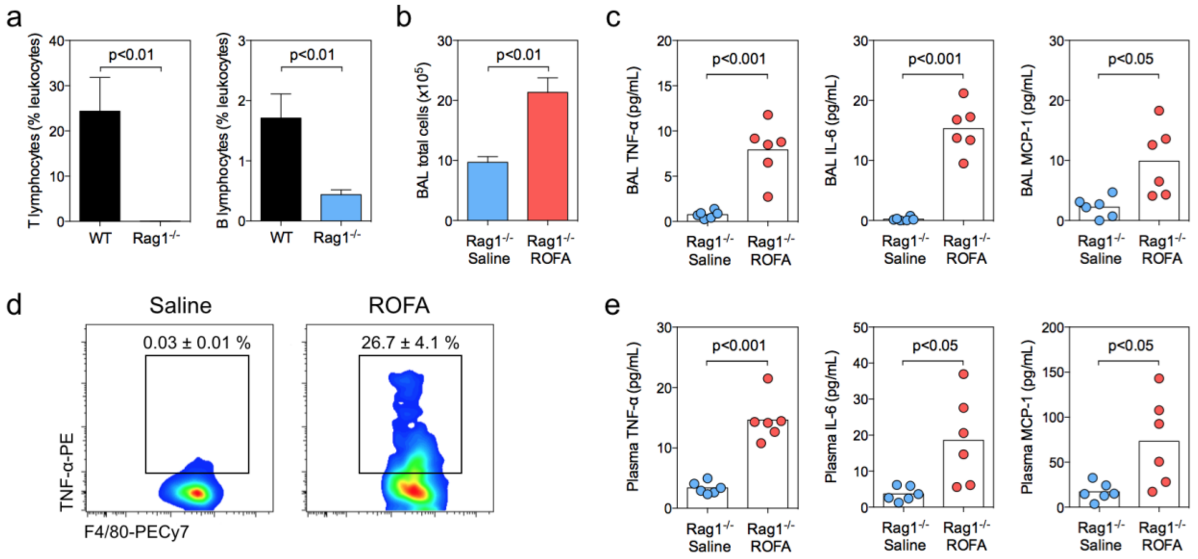

Supplementary Fig. 8: Lymphocytes are not required for ROFA-mediated cytokine secretion. $Rag1^{-/-}$ mice lack mature T- and B- lymphocytes in peripheral blood as confirmed by flow cytometry (a). $Rag1^{-/-}$ mice were exposed to saline solution or ROFA particles (1 mg/kg body weight) by i.n. instillation. After 3 hours, total cell numbers (b) and levels of pro-inflammatory cytokines (c) were evaluated in BAL samples. TNF-α expression was quantified by intracellular staining in alveolar macrophages from BAL cell suspensions (d). Plasma levels of pro-inflammatory cytokines were quantified by a cytometric bead array (e). Data are presented as mean ± SEM of at least 6 mice per group.

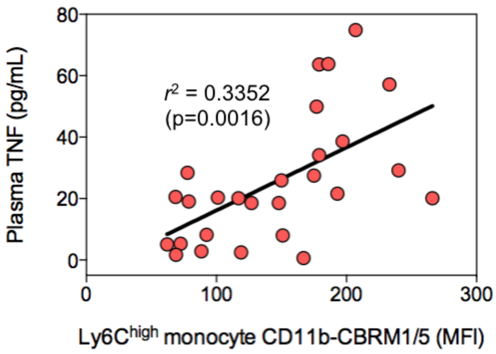

Supplementary Fig. 9: CD11b activation on blood inflammatory Ly6C^{high} monocytes correlates with plasma TNF-α levels. Linear correlation of CD11b-CBRM1/5 expression (expressed as MFI) on inflammatory Ly6C^{high} monocytes and TNF-α levels in blood samples from ROFA-exposed mice after LAD ligation.

SUPPLEMENTARY VIDEOS LEGEND

Supplementary Videos I and II demonstrate how leukocyte rolling and adhesion is altered in mesenteric vessels by an i.n. instillation of saline solution (Supplemental Video I) or ROFA particles (Supplemental Video II). For a positive control, mice were injected i.p. with 200 ng TNF-α (Supplemental Video III). Leukocyte recruitment was imaged 4 hours later. Supplementary Videos IV and V shows leukocyte rolling and adhesion in mesenteric vessels following an i.n. instillation of saline solution (Supplemental Video IV) or ROFA particles (Supplemental Video V) in mice pretreated with i.n. PBS liposomes. Supplemental Video VI shows leukocyte trafficking in i.n. clodronate-pretreated ROFA-exposed mice.

SUPPLEMENTARY REFERENCES

1. Chen Y, Shah N, Huggins FE, Huffman GP (2004) Investigation of the microcharacteristics of PM2.5 in residual oil fly ash by analytical transmission electron microscopy. Environ Sci Technol 38:6553-6560 doi: 10.1021/es049872h
2. Ghio AJ, Silbajoris R, Carson JL, Samet JM (2002) Biologic effects of oil fly ash. Environ Health Perspect 110 Suppl 1:89-94
3. Hilgendorf I, Gerhardt LM, Tan TC, Winter C, Holderried TA, Chousterman BG, Iwamoto Y, Liao R, Zirlik A, Scherer-Crosbie M, Hedrick CC, Libby P, Nahrendorf M, Weissleder R, Swirski FK (2014) Ly-6Chigh monocytes depend on Nr4a1 to balance both inflammatory and reparative phases in the infarcted myocardium. Circ Res 114:1611-1622 doi:10.1161/CIRCRESAHA.114.303204
4. Huggins FE, Galbreath KC, Eylands KE, Van Loon LL, Olson JA, Zillioux EJ, Ward SG, Lynch PA, Chu P (2011) Determination of nickel species in stack emissions from eight residual oil-fired utility steam-generating units. Environ Sci Technol 45:6188-6195 doi:10.1021/es200823a
5. Nurkiewicz TR, Porter DW, Barger M, Castranova V, Boegehold MA (2004) Particulate matter exposure impairs systemic microvascular endothelium-dependent dilation. Environ Health Perspect 112:1299-1306

6. Nurkiewicz TR, Porter DW, Barger M, Millecchia L, Rao KM, Marvar PJ, Hubbs AF, Castranova V, Boegehold MA (2006) Systemic microvascular dysfunction and inflammation after pulmonary particulate matter exposure. Environ Health Perspect 114:412-419
7. Ostachuk A, Evelson P, Martin S, Dawidowski L, Sebastian Yakisich J, Tasat DR (2008) Age-related lung cell response to urban Buenos Aires air particle soluble fraction. Environ Res 107:170-177 doi:10.1016/j.envres.2008.01.007
8. Pattanaik S, Huggins FE, Huffman GP (2012) Chemical speciation of Fe and Ni in residual oil fly ash fine particulate matter using X-ray absorption spectroscopy. Environ Sci Technol 46:12927-12935 doi:10.1021/es301080s
9. Southam DS, Dolovich M, O'Byrne PM, Inman MD (2002) Distribution of intranasal instillations in mice: effects of volume, time, body position, and anesthesia. Am J Physiol Lung Cell Mol Physiol 282:L833-839 doi:10.1152/ajplung.00173.2001
10. van Rooijen N, Hendrikx E (2010) Liposomes for specific depletion of macrophages from organs and tissues. Methods Mol Biol 605:189-203 doi:10.1007/978-1-60327-360-2_13
11. Wolf D, Hohmann JD, Wiedemann A, Bledzka K, Blankenbach H, Marchini T, Gutte K, Zeschky K, Bassler N, Hoppe N, Rodriguez AO, Herr N, Hilgendorf I, Stachon P, Willecke F, Duerschmied D, von zur Muhlen C, Soloviev DA, Zhang L, Bode C, Plow EF, Libby P, Peter K, Zirlik A (2011) Binding of CD40L to Mac-1's I-domain involves the EQLKKSKTL motif and mediates leukocyte recruitment and atherosclerosis--but does not affect immunity and thrombosis in mice. Circ Res 109:1269-1279 doi:10.1161/CIRCRESAHA.111.247684
12. Zasłona Z, Przybranowski S, Wilke C, van Rooijen N, Teitz-Tennenbaum S, Osterholzer JJ, Wilkinson JE, Moore BB, Peters-Golden M (2014) Resident alveolar macrophages suppress, whereas recruited monocytes promote, allergic lung inflammation in murine models of asthma. J Immunol 193:4245-4253 doi:10.4049/jimmunol.1400580
